# Annual Report of the Section on Obstetrics and Diseases of Women and Children of the Chicago Society of Physicians and Surgeons

**Published:** 1873-03

**Authors:** A. Reeves Jackson

**Affiliations:** Chairman of the Section


ARTICLE II.—*Annual Report of the Section on Obstetrics and Diseases of Women and Children of the Chicago Society of Physicians and Surgeons.* DEPARTMENT OF GYNÆCOLOGY. Read before the Society, December 9, 1872, by A. REEVES JACKSON, M.D., Chairman of the Section.

In the report on Diseases of Women which I have the honor to present to the Society, I have endeavored to bring together such subjects of interest and importance as have appeared during the present year in the various periodicals to which I have had access, with the view of showing, so far at least as the current literature represents it, the existing state of the science. In doing this, I have refrained from introducing any comments of my own, believing that the Society would much prefer having the opinions of those who are now prominent in our own and other countries in this department of practice.

Neither has there been any attempt at systematic arrangement of subjects. I have taken them just as they occurred to me, and have done nothing more than simply group together such as were of the same or a similar character.

### DILATATION OF THE OS AND CERVIX UTERI.

Dr. Atthill, in Braithwaite's Retrospect for January, 1872, says, "Whenever you meet with a case of menorrhagia in a woman who is *otherwise unhealthy*, which a careful vaginal examination proves not to depend on ulceration of the os and cervix uteri, or an extra-uterine polypus, or cancer, or such evident cause, it is a manifest duty to dilate the cervix and os internum with the view of determining the condition of the interior of the womb."

Of the two methods of doing this, viz., the one by the use of sponge tents and the other by means of the sea tangle, he prefers the latter. The mode adopted by him is the one first introduced by Dr. Kidd, of Dublin. It consists, first, in exposing the os uteri by means of the duck-bill speculum, and drawing down the uterus with a small hook inserted into the anterior lip. Several pieces of sea tangle bougie, each cut the full length of the uterine cavity, are then to be inserted separately side by side. These are to remain from twelve to twenty-four hours.

In case this does not produce so much dilatation as may be desirable, the process may be repeated, and if this should also fail, the dilatation may be completed by the use of one of Barnes' dilators.

Dr. Atthill condemns in strong terms the use of any of the metallic instruments which have been suggested for the purpose of dilating the cervix, and asserts that their use is attended with danger, as they act too rapidly and sometimes rupture the uterine fibres, several cases of severe inflammation and even of death having followed their use, while sea tangle is perfectly harmless.

Dr. Grailly Hewitt still prefers the sponge tents for the purpose of dilating the uterine cavity, objecting to the laminaria tents, that they are liable to slip out of place.

MECHANICAL DYSMENORRHŒA.

Dr. Atthill (Braithwaite, Jan., 1872,) states, that there are three varieties of mechanical dysmenorrhœa: 1. That in which the cervical canal is so flexed as to obstruct the escape of the menstrual discharge; 2. That in which inflammation or congestion of the lining membrane exists to such an extent as to cause temporary closure of the canal; 3. That in which there is some congenital narrowing of the os internum of the cervical canal.

He regards the operation of incising the os internum and the cervix, first introduced by the late Prof. Simpson, as proper in the treatment of all these varieties, after the failure of other means, including the dilatation of the canal by the use of sea tangle tents. For the performance of the operation, he gives preference to the instrument of Dr. Savage. It is furnished with two blades, the cutting edge of each being directed outward, but as the back of each blade, when the instrument is closed, projects beyond the edge of its fellow, which it thus overlaps, its introduction into the cervix can be safely effected; but it is generally necessary to dilate the cervical canal before this step can be effected. This is done by the insertion of sea tangle tents, two of which will commonly be sufficient for this purpose. In accordance with the opinion of most gynæcologists, Dr. Atthill limits the use of the hysterotome to the division of the os internum and the supra-vaginal portion of the cervix, and prefers dividing the vaginal portion with a pair of curved scissors.

In order to prevent the incisions from uniting, to which there is sometimes a great tendency, he mentions the suggestion of Dr. Coglan to insert a thin roll of lead, and of Dr. Grailly Hewitt to introduce an ebony stem pessary. The last named means would, in addition to preventing contraction, also tend to keep the canal straight.

### DYSMENORRHŒA AND STERILITY.

Dr. J. Protheroe Smith, (Braithwaite, July, 1872.)

In certain cases of mechanical dysmenorrhœa, there exists simply a stricture of the os internum, with narrowing of the cervical canal and mouth. All accompanying congestion or inflammation being previously removed, Dr. Smith recommends forcible extension by means of a dilator. After preparing the patient by a purgative dose, and by abstinence from all local excitement, and from alcoholic drinks, or much animal food, the uterine canal is accustomed to bear a metal bougie, which should be repeatedly and daily introduced, and increased in size until that of a No. 10 catheter can be borne without any pain. Then the uterine dilator may be safely employed. The instrument he employs consists of two short blades, two and a half inches long, separated by a screw worked with a nut, so as to mark precisely by an index on the handle, the extent of the dilatation employed. This should be used at first cautiously about every second day, always ceasing to turn the screw so soon as pain is experienced. In the course of a few days or weeks at farthest, a forced dilatation to the extent of an inch or an inch and a half may be made with impunity. After this the dilator should be used daily for two or three days, and afterwards at longer intervals, to keep the parts open till they permanently heal in the state of distension effected by the operation.

The results of this treatment are more permanent than that by division of the constriction by means of the hysterotome, which although it gives prompt temporary relief, sometimes produces a firm cicatricial constriction, which is worse than the original disease.

### TREATMENT OF SOME FORMS OF MENORRHAGIA.

Dr. Atthill, (Braithwaite, Jan., 1872.)

In cases of menorrhagia, depending on sub-involution of the uterus after labor or abortion after the failure of ergot, gallic acid and similar remedies, we should introduce ten grains of the solid nitrate of silver as high as the fundus of the uterus, and allow it to remain there and dissolve.

Dr. Atthill effects this object by means of the instrument known as Simpson's *uterine porte caustique;* an instrument which consists of a hollow silver tube open at both ends, in size and shape closely resembling a sound; it contains an accurately fitting flexible stilet. The instrument is introduced to the fundus, the stilet is then withdrawn and the piece of solid nitrate of silver, reduced to the proper size and shape, placed in the tube. It is pushed upwards by means of the stilet until it reaches the end of the tube. This latter is then withdrawn about half an inch in order to guard against the possible forcing of the nitrate of silver into the uterine tissues. This having been done, the caustic is pushed out of the instrument and left free in the cavity of the womb. Dr. Atthill has used this practice several years, and considers it simple, safe and efficient. It produces some pain for a few hours, but he has known no further unpleasant results.

Sometimes a severe form of menorrhagia results from that unhealthy, spongy condition of the os and cervix, in which the mucous membrane lining the uterine canal becomes hypertrophied and thickened, the os uteri patulous, and the lips everted. In order to effect a cure in these cases it is necessary that our treatment should reach every portion of the diseased tissue, which usually extends, at least, as high as the os internum. Two pieces of sea tangle should therefore be introduced and permitted to remain twenty-four hours, at the end of which time they should be withdrawn, and the whole diseased surface should then be cauterized with fuming nitric acid. In a case cited, this caused no pain. In a few days the parts, on being examined, were found in a much healthier state. *The menorrhagia was entirely checked and never returned.* The subsequent treatment consisted in the occasional application of a twenty-grain solution of nitrate of silver to the os uteri, and, at a later period, of a small blister to the sacrum. Finally, the ulceration was entirely removed, and menstruation became in all respects normal.

### MENORRHAGIA AND METRORRHAGIA.

J. Matthews Duncan, (Braithwaite, July, 1872.)

Treatment is divided into that which is proper before the loss, and that which is proper while the loss is going on.

The first consists in the use of remedies for any condition which may appear to predispose to, or aid in maintaining the complaint. The second is the chief treatment, and is various in kind. The patient should rest in the horizontal position, and be kept cool. This is applicable to all cases. The application of cold cloths to the abdomen, vulva, etc., or cold water thrown into the vagina or rectum, is a remedy of doubtful value. The vaginal plug, consisting of compressed sponge or bits of lint saturated with alum or perchloride of iron, is sufficient to check the bleeding in most cases. In extreme cases the mucous membrane of the cavity of the uterus may be painted with solution of perchloride of iron. There is nothing worthy the name of evidence to show that the internal administration of the so-called hemostatic remedies, as the mineral acids, ergot, digitalis, gallic and tannic acids, has any considerable effect in checking the flow, but they may be used for the possible good they may do. Ten to twenty drops of the dilute sulphuric acid in infusion of roses, four to eight times daily, is one of the best.

### MENORRHAGIA.

J. H. Aveling, (Braithwaite, July, 1872.)

In cases of menorrhagia and leucorrhœa, depending upon simple hyperemy—a word invented by Andral, and defined by him to signify an excess of blood in the capillaries—we should administer arsenic in the form of Fowler's solution, in doses of from two to six drops, three times a day with meals. The dose should be two drops to commence with, and should be continued a fortnight. If at the end of that time no conjunctival irritation should display itself, the dose may be increased to four drops; and then again, after another interval, to six, eight or ten, or even more drops.

The first effect of this treatment is to improve the digestive powers, and the patient's general appearance. The nervous system shows increased tone. The intercatamenial period is gradually lengthened, the amount of the discharges is decreased, and in like manner all the other hyperæmic symptoms disappear.

### TREATMENT OF THE PEDICLE IN OVARIOTOMY.

Dr. D. Lloyd Roberts, (Braithwaite, January, 1872.)

No one method of securing the ovarian pedicle is of universal application. If it is long, narrow, and can be easily brought outside without traction upon the uterus, or making any undue pressure upon the wound at the lower part of the belly, we should use the clamp; if, on the contrary, the pedicle is short, and there is danger of dragging or too much displacement of the uterus; and if it is not too voluminous, it should be transfixed with a firm ligature, crossed so that it may be tied on one side only, the ligature cut close, and the stump dropped into the pelvis. In cases where the pedicle can neither be safely brought out with the clamp, nor ligatured for fear of vessels retracting or tissues shriveling up; or where the pedicle is very short, vascular and fleshy; or where it is very close to the uterus, it should be secured with a clamp (moderately crushing it), divided, each vessel secured with a ligature, cut short, and the actual cautery applied to the rest of the stump, waiting a few minutes to see if any oozing of blood take place before dropping it into the pelvis. Should the pedicle be very voluminous, the cyst having a very broad attachment to the uterus, so that the clamp cannot be used to hold and crush it while the cautery is being applied, it should be slowly divided with a pair of blunt scissors, each vessel being taken up as it is opened, and the cautery applied to the remaining portion of the stump.

To tie and return the pedicle seems to be the next best mode of dealing with the stump, when, owing to its shortness, the clamp cannot be used. The portion of the pedicle on the distal side of the ligature, surrounded as it is by *warm* tissues, retains its vitality long enough for it to become attached by lymph to the adjacent parts. The ligature, when tightened, buries itself too, and brings into apposition the peritoneal covering on each side of it, and between these adhesion soon takes place.

### RETROFLEXION OF THE UTERUS.

Dr. Robert Barnes, (Braithwaite's Retrospect, Jan., 1872.)

Dr. Barnes recommends the treatment of retroflexion by means of the lever pessary, which should be in preference made of vulcanite. The size of the instrument should not be so great as to put the posterior vaginal wall on the stretch, otherwise it will produce pain, and lose the character which a lever should have, of mobility. Any accompanying uterine disease should at the same time receive its appropriate treatment.

UTERINE POLYPI.

Dr. J. Matthews Duncan, (Braithwaite's Retrospect, Jan., 1872.)

Uterine polypi are of three kinds, fibrous, fibrinous, and mucous. The latter are the most frequent.

The constancy of hemorrhage as a symptom of this disease has been greatly exaggerated. In forty-one cases of mucous polypi, nineteen caused no extraordinary loss of blood, while in several there was less than ordinary loss, and in some no discharge of any kind.

The largest losses of blood are produced by true fibrous polypi. In only one of twenty-two fibrous polypi, was there no extraordinary loss of blood.

When the existence of polypus is suspected, and a vaginal examination does not reveal it, it will be necessary to dilate the os uteri. It is very rarely necessary to dilate the whole cervix, this proceeding having been required in only one of sixty-six cases. Dilatation is effected by means of a sea tangle tent, succeeded in twenty-four hours by a larger one, and this again by one still larger, if necessary. The tent, with a string attached to its lower end, to facilitate removal, is seized by a vaginal or throat forceps, and passed into the uterus. In order to keep it *in situ* it is well to introduce a small sponge into the vagina. After the removal of the tent, the index finger of one hand is passed into and through the os uteri, while the other hand, pressing on the abdomen above the pubes, pushes the womb downwards upon the examining finger.

There is nothing simpler than to tell what should be done in a case of polypus. It should be removed.

Mucous polypi, when small, may be removed by torsion, it, or its pedicle, being seized by appropriate forceps and twisted until it separates. If too large for this, and it exceeds the phalanx of a small finger, it is best to clip through the pedicle with curved scissors. The operation may be done without a speculum. Neither is an assistant necessary, although it is better to have one.

The various instruments that have been invented for cutting through the pedicle of a polypus are unnecessary. There is no difficulty in dividing the pedicle by means of a bistoury or curved scissors—the difficulty consists in reaching the pedicle. In cases of enormous polypi, there is difficulty in delivery from the vagina. The perineum is likely to be torn, and sometimes the laceration extends even through the sphincter ani. Division of the pedicle, even if it could be effected as a preliminary to the operation, would not make any essential difference in this respect.

In these cases Dr. Duncan removes portion after portion of the tumor, in such a way, if possible, as always to leave a convenient bit of the tumor projecting from the remaining part, by which a good hold is got for pulling the tumor further down. So, bit by bit, the whole is removed.

### FIBROID TUMORS AND POLYPI.

Dr. Thomas Skinner, (Braithwaite's Retrospect, July, 1872.)

To facilitate removal, the first step is to open up the passages for the introduction of the necessary instruments, and to attain greater certainty as to the position, relations and attachments of the tumor and its pedicle. This is best accomplished by means of sea tangle and sponge tents, and, if necessary, by incising the cervix uteri.

Next, the tumor is to be fixed by means of a vulsellum, or Dr. McClintock's corkscrew, and then transfixed with a cord, or by placing a noose over it. The tumor being firmly grasped, is pulled as low as it will admit of, and the wire or chain of the ecraseur passed over it to its pedicle. In case this latter is so thick and fibrous that it cannot be divided in this manner, our next best step is to divide it immediately above the wire or chain of the ecraseur with blunt-pointed curved scissors, a blunt-pointed curved bistoury, or, best of all, with the polyptome of Sir James Simpson.

In cases where, from any cause, the whole of a fibrous tumor or a fibroid polypus cannot be removed, it is good practice to remove as much of it as possible, as the remainder will frequently thereafter disappear.

The instruments that may be necessary in the removal of fibroid tumors are the following, namely: the uterine sound; wire and chain ecraseur; Simpson's vulsellæ, large and small; Simpson's polyptome; intra-uterine scissors; strong blunt-pointed scissors, curved, for dividing the os and cervix; small uterine syringe, for hemostatic injection; uterine scrapers; polypus crusher; small, narrow midwifery forceps; sponge and laminaria tents, and their guides or introducers; wire and other ligaturing material; sponges; an ordinary pocket case of instruments.

FIBROID TUMORS OF THE UTERUS.

Dr. Alfred Meadows, (Braithwaite's Retrospect, July, 1872.)

Fibroid tumors of the uterus present almost the same histological elements as the uterus itself, viz., smooth or unstriped muscular fibres, bound together with varying quantities of connective tissue.

These tumors are usually not very painful, unless their size be such as to cause pressure on the neighboring organs, or are so situated as to project prominently from the peritoneal surface. A small tumor in this position will often occasion very great suffering, while a patient may have a very large one growing into the uterine cavity, with little or no pain. The pain and hemorrhage are generally in inverse proportion the one to the other, the subperitoneal producing the most pain and the least hemorrhage, while the submucous and interstitial tumors occasion the most hemorrhage and the least pain.

The pain in these cases is best relieved by medicated vaginal pessaries; but inasmuch as the vaginal mucous membrane has no power to absorb fats, cocoa-butter should not be used as the excipient in their preparation. It is much better to use gelatine and glycerine as the basis of the pessary, in the proportion of one part of the former to four of the latter; into this we can put any ingredient we wish, as atropine, morphia, conia, or any other agent of this class.

The hemorrhage is, however, the symptom which we shall most frequently be called upon to treat, and the treatment is, as a rule, most unsatisfactory and disappointing. Ergot, upon the whole, excels in hemostatic properties in these cases. It is more useful where the tumor is interstitial than where it is submucous; it fails, therefore, not unfrequently in the very cases where it is most needed, for it is in the submucous varieties that we get the greatest amount of hemorrhage. Usually it is best to combine the ergot with some purely astringent substance, and when anemia is present, some form of iron. In case it becomes necessary to resort to topical applications, the most effective is the solid stick of anhydrous sulphate of zinc. This is preferable to fluid injections into the uterus, being fully as useful and far less dangerous. It should be passed through the speculum, quite up into the uterine cavity. Dr. Meadows has frequently tried the plan of Dr. Baker

Brown, of freely incising the cervix, for the purpose of curing the hemorrhage, but has not seen any good result from it.

### FIBROUS TUMORS OF THE UTERUS.

Dr. L. Atthill, (Braithwaite's Retrospect, Jan., 1872.)

A fibrous tumor may be defined to be a growth composed of fibrous tissue identical in structure with that of the uterine wall, but disconnected from it, being, in general, surrounded by a capsule of dense fibro-cellular tissue, which is perfectly dry and loose, so that when one cuts down on the tumor it almost of itself escapes from the cavity. There are three classes, namely: subperitoneal, submucous, and intra-mural, according as they are found to grow from the peritoneal surface of the uterus, from its submucous tissue, or are developed within the walls of the organ.

The first, or subperitoneal, are beyond the reach of treatment. They do not give rise to menorrhagia, and indeed do not seem as a rule to influence menstruation at all.

The submucous, pedunculated, fibrous tumor is to be treated in a manner identical with the ordinary fibrous polypus.

The intra-mural are of frequent occurrence, and the most important. They nearly always produce menorrhagia, and as invariably stimulate the uterus to enlargement. Medicines without number have been employed with the view of causing absorption of fibrous tumors of the womb. Prominent among these are the bromides. After a full trial, Dr. Atthill has found little good result from their use, and has lost all faith in their resolvent powers in the disease under consideration.

The surgical means that have been recommended and used for their removal are five in number. They are—1. Incising the cervix uteri; 2. Incising the tumor; 3. Cutting into the tumor and destroying a portion of its tissue—a process to which the term gouging has been applied; 4. Enucleation of the tumor; 5. Avulsion, or the forcible tearing away of the tumor from its attachment.

Each of these methods has been used with various degrees of success. The operation of incising the os is founded on the theory of Dr. Baker Brown, "that the division of the os and cervix uteri permits the fibres of the body of the uterus to contract upon the contained tumor, and thereby to compress the vessels and prevent hemorrhage." Whether this be the true explanation or not, it is certain that the operation is often followed by good results, and in the case of large tumors which are contained within the uterus, and where the cervix is thinned and spread over them, the operation is fully justified. The incising of the tumor has been practiced by Dr. Atlee, of Philadelphia, and Dr. Tracy of Melbourne, and others, with a success which is probably due to the fact that the vitality of these tumors is nearly, if not altogether, destroyed by the incision having divided their capsules, for the fibrous growth itself is endowed with a very low degree of vitality.

Enucleation is practiced by Dr. Matthews Duncan and others, and this gentleman has also practiced with great success the method of avulsion. This consists in seizing the tumor with a strong vulsellum, and forcibly dragging it from its attachments. It is specially suitable for those cases in which spontaneous enucleation has already partially begun, or where that stage having been artificially commenced, has proceeded to some extent.

Dangerous hemorrhage, from the pressure of a fibrous tumor, may be checked by injecting into the uterine cavity—the cervix having been previously dilated—the tincture of iodine or a solution of perchloride of iron.

### CANCER OF THE WOMB SUCCESSFULLY TREATED BY BROMINE.

Dr. A. Wynn Williams, (Braithwaite, Jan., 1872.)

Dr. Williams gives the details of four cases of medullary and epithelial cancer of the cervix successfully treated by the injection and application of bromine, twelve drops to one dram rectified spirits of wine. His opinion is, that cancerous growths or formations situated in the lips or neck of the womb can be successfully medicated when confined to this part only, and previous to the commencement of ulceration. The presence of ulceration, indeed, should not preclude an attempt at removal so long as the womb is movable, and the neighboring glands and other parts remain apparently healthy. The mode of proceeding will vary in different cases. The more superficial, as some of the epithelial varieties, will be best destroyed by saturating cotton wool with the bromine solution, placing over it a vulcanite cup or gutta percha. Any stump left must be treated by injection, as the more solid tumors.

The solid growths are best treated by injection of their substance, and for this purpose a small trocar and canula, to which a glass syringe can be attached, answers best.

Dr. Williams' paper, detailing the cases in which he had used this treatment, was read before the Obstetrical Society of London, and was freely criticised, especially by Dr. Playfair, who remarked that Dr. Williams' cases afforded no conclusive proof that they were malignant at all, inasmuch as fixature of the womb, which is the first positive sign of malignancy, did not exist; indeed, in each instance the womb was said to be freely movable. Dr. Williams stated, in reply, that there must be a time in the early stages of the disease, when it was confined solely to the neck of the uterus, and in which the womb was not fixed; such was the case in the mammæ and other organs.

### CARCINOMA OF THE UTERUS.

Dr. O. Spiegelburg, (Half Yearly Compendium, July, 1872.)

Dr. Spiegelburg states, that the diagnosis of carcinoma in its first stage may be made with tolerable certainty by the introduction of a sponge tent. If the hardness of the os be owing to a benign tumor or infiltration, it will allow of dilatation; but if it be owing to malignant disease, the tissue will remain rigid and inelastic, even after the sponge has been allowed to remain twenty-four hours.

He recommends the early removal of the cervix, believing that occasionally, though rarely, radical cures are thus obtained. Where the operation cannot be performed at a very early period, he advises merely a symptomatic treatment.

### INFUSION OF TOBACCO IN VAGINITIS.

Dr. L. Atthill, (Half Yearly Compendium, Jan., 1872.)

Infusion of tobacco is a valuable remedy in vaginitis, but in using it we should be sure that the orifice of the canal is sufficiently patulous to permit the free escape of the fluid. In some cases where the vaginal orifice is small, the use of the remedy is quickly followed by faintness, nausea, etc. The value of the remedy is greatly enhanced by the addition of two or three drams of borax.

### ADVANTAGE OF ELEVATING THE WOMB.

Dr. Cumming, (Half Yearly Compendium, Jan., 1872.)

Dr. Cumming states that sufficient importance has not been assigned to the maintenance of the womb at its proper height. A descent of one inch often produces distressing sensations, and in married women exposes the organ to injury during the sexual act—resulting in tenderness, pain, swelling and ulceration.

For the purpose of elevating the womb, he advises the use of the ring pessary, of as large a size as can be borne. The ring should be compressed into an elliptical shape by tying firmly with tape, a slip knot being used. After introducing the pessary, the knot may be loosened by pulling on the free end. The ring will then resume its original shape, and may then be finally adjusted. Many ulcerations of the cervix get well under this treatment without any local application whatever.

### ON THE USE OF CLOTH TENTS.

Dr. Talliaferro, (Atlanta Medical and Surgical Journal for Oct., 1871), publishes an article on the use of cloth tents. In his experience, he had found the sponge and sea tangle too irritating, while from the cloth tents he had found all the good results of the others without the evils.

Cloth tents can be medicated to meet the indication for any case or condition. For *dilating*, their continued use, increasing the size from day to day, they are preferable to other tents, especially where the mucous surface is diseased.

They are prepared by rolling a strip of cloth, of the desired width, firmly between the finger and thumb, regulating the size and length by distance of lap in the roll, and length of cloth rolled into one tent, the strips being about one inch wide.

### THE RECUMBENT POSTURE IN THE TREATMENT OF UTERINE DISPLACEMENTS.

Dr. Wm. L. Edgar, (St. Louis Medical Archives, Aug., 1872.)

Dr. Edgar insists upon the importance of rest in bed, in the treatment of displacements, and attributes one-half of the failures to cure these ailments, to neglect of this all-important precaution.

